# Cyclic Peptide Inhibitors of the Tsg101 UEV Protein Interactions Refined through Global Docking and Gaussian Accelerated Molecular Dynamics Simulations

**DOI:** 10.3390/polym12102235

**Published:** 2020-09-28

**Authors:** Wen-Wei Lin, Yu-Jen Wang, Cheng-Wen Ko, Tain-Lu Cheng, Yeng-Tseng Wang

**Affiliations:** 1 School of Post-Baccalaureate Medicine, College of Medicine, Kaohsiung Medical University, Kaohsiung 807, Taiwan; wwlin0627@kmu.edu.tw; 2 Drug Development and Value Creation Research Center, Kaohsiung Medical University, Kaohsiung 807, Taiwan; tlcheng@kmu.edu.tw; 3 Graduate Institute of Medicine, Kaohsiung Medical University, Kaohsiung 807, Taiwan; 4 Department of Mechanical and Electromechanical Engineering, National Sun Yat-sen University, Kaohsiung 804, Taiwan; yjwang@mail.nsysu.edu.tw; 5 Department of Computer Science and Engineering, National Sun Yat-sen University, Kaohsiung 804, Taiwan; cwko@cse.nsysu.edu.tw; 6 Department of Medical Research, Kaohsiung Medical University Hospital, Kaohsiung 80756, Taiwan

**Keywords:** molecular dynamics, Tsg101 UEV protein, Gaussian accelerated molecular dynamics simulation, cyclic peptides

## Abstract

Tsg101 UEV domain proteins are potential targets for virus infection therapy, especially for HIV and Ebola viruses. Peptides are key in curbing virus transmission, and cyclic peptides have a greater survival time than their linear peptides. To date, the accurate prediction of cyclic peptide-protein receptors binding conformations still is challenging because of high peptide flexibility. Here, a useful approach combined the global peptide docking, Gaussian accelerated molecular dynamics (GaMD), two-dimensional (2D) potential of mean force (PMF), normal molecular dynamics (cMD), and solvated interaction energy (SIE) techniques. Then we used this approach to investigate the binding conformations of UEV domain proteins with three cyclic peptides inhibitors. We reported the possible cyclic peptide-UEV domain protein binding conformations via 2D PMF free energy profiles and SIE free energy calculations. The residues Trp145, Tyr147, and Trp148 of the native cyclic peptide (CP1) indeed play essential roles in the cyclic peptides-UEV domain proteins interactions. Our findings might increase the accuracy of cyclic peptide-protein conformational prediction, which may facilitate cyclic peptide inhibitor design. Our approach is expected to further aid in addressing the challenges in cyclic peptide inhibitor design.

## 1. Introduction

Human Tsg101 proteins are important receptor proteins required for budding of the enveloped viruses (HIV, RSV, HSV-1/2, Ebola and parainfluenza) [1,2,3,4]; thus, protecting the release of virions from an infected cell is crucial [5,6]. Tsg101 proteins are recruited to the virus budding sites by binding to the PT/SAP motifs located within the HIV Gag p6 region and the Ebola Vp40 proteins, and then Tsg101 proteins and other cellular factors complete the budding process [2]. Inactivation of the Tsg101-expressing gene [5] through mutation or deletion results in the budding process failure of HIV or Ebola virus. Thus, pharmacological disruption of Tsg101–PT/SAP motifs interactions can stop to release immature virions form these infected cells. The *N*-terminal ubiquitin E2 variant (UEV) domain of Tsg101 proteins can bind to the PT/SAP motifs on HIV Gag p6 region or Ebola Vp40 proteins [7,8], whereas the remaining C-terminal part of Tsg101 UEV has an autoinhibitory function [6]. While the UEV domain proteins are important therapeutic protein targets, there are no efficiently drugs that can inhibit HIV and Ebola budding.

Esomeprazole and tenatoprazole are reported that the two small molecules can inhibit the p6/UEV interactions may be applicable in HIV and Ebola virus infection therap [9]. However, the lowest effective concentration (EC50) of the two inhibitors are 25–50 μM, and the two inhibitors are not enough to against P/UEV interactions [9]. The detail interactions of the p6 region of HIV Gag and the Ebola Vp40 to UEV domain proteins regulation warrant further exploration, but these small molecules inhibitors are not optimized in highly expressing UEV protein levels [9]. Despite the structural similarity between UEV and P6 motifs, the conformational diversity of UEV-binding region makes the development of small compound inhibitors challenging.

A linear peptide with a low affinity of 54 uM, the wild-type synthetic peptide (amino sequence: PEPTAPPEE), was reported as an inhibitor of UEV for HIV and Ebola virus infection therapy [10]. However, linear peptides have a short life in mammalian blood, partly because of *enzymatic degradation* within minutes. From the structural and pharmacological standpoint, cyclic peptides show higher life time and better biological activity compared with linear form peptides [11]. Cyclic peptides are polypeptide chains taking ring structures, wherein the peptides are formed by linking the one and end of the peptides with amide bonds or other covalent bonds. Cyclic peptides found in natue or synthesized in the laboratory are used in clinic. A cyclic peptide (Table 1) [12] was reported to efficiently inhibit the UEV domain of Tsg101. Our research investigated novel cyclic peptide inhibitors for HIV and Ebola virus infection therapy.

Peptide–protein docking remains a great challenge for most computational docking programs because of the peptide flexibility [13,14]. Small chemical molecules usually can strongly interact with small binding motifs and grasp the motifs in protein receptors. Except GPCR receptors, the peptides normally interact with the largest pockets and only bind to the protein receptors surface. Therefore, most peptides and protein receptors can’t form stable complex structures [15]. There are template-based, local and global methods in regard to the peptide-protein docking simulations [14]. Since the template-based and local peptide docking methods require the 3D structural binding information, these approaches must be tested and verified by qualifying with the Critical Assessment of Prediction of Interactions criteria (CAPPRI) [16,17]. These approaches usually have a high performance but are often limited by the availability of experimental 3D structure information. The global peptide docking is that free peptides bind to protein receptors without experimental 3D structure information, but it is challenging to account for the protein receptors and peptides conformational flexibility. The ClusPro docking is a fast and global peptide docking method [18,19]. Unfortunately, this method still cannot provide more accurate 3D structural predictions of the peptide binding [20]. To address this issue, the approach combined ClusPro and Gaussian accelerated MD (GaMD) methods have been successfully tested [13]. The combined approach can refine the complex structures and significantly reduced the linear peptide backbone RMSDs to 0.6–2.7 Å. We also followed our strategy to simulate the refinement of the Tsg101 UEV protein and the linear peptide (amino sequence: PEPTAPPEE). Then our refinement was compared with the x-ray structure (PDB ID: 3obu). Our results are shown in Figures S1–3. In this study, we used molecular simulation techniques to investigate the interactions of UEV domain protein with these cyclic peptides (N to C terminal, Table 1) with the approach combined the ClusPro and GaMD methods. For verifying our predictions, the solvated interaction energy (SIE) free energy calculations were performed, and our predicted free energies were comparied with the experimental binding free energies(Table 1) [21]. Our simulations provide further insight into the binding interactions between UEV domain protein and cyclic peptides.

## 2. Materials and Methods

We followed Miao et al.’s approach, and the ClusPro docking, normal MD, GaMD, and binding free energy calculations were performed in the cyclic peptide-UEV domain protein receptor calculations [13]. This strategy consists of the following steps:

Step 1: Building and optimizing the cyclic peptides by using the EVGAZZ software.
Step 2: Docking the cyclic peptides to UEV domain protein receptor by using ClusPro software.
Step 3: Selecting possible cyclic peptide-UEV domain protein receptor complex structures from step 2.
Step 4: Performing energy minimizations, NTV (1ns), and NPT (1ns) equilibrium simulations by using pmemd.cuda (Amber18).
Step 5: Performing 20-ns GaMD equilibrium simulations by using pmemd.cuda (Amber18).
Step 6: Performing 300-ns GaMD production simulation four times by using pmemd.cuda (Amber18).
Step 7: Performing analysis of reaction coordinates by using AmberTools cpptraj software to obtain 2D potential of mean force profiles (2D PMF) with PyReweighting toolkit.
Step 8: The complex structures with the lowest potential of mean force (PMF) values were performed in 10-ns cMD simulations for SIE free energy calculations and binding mode analysis [22].

### 2.1. Cyclic Peptide Building

The three cyclic peptides (Table 1) were built on VEGAZZ. UEV domain protein crystal structures (PDB ID: 3obu) were selected as receptors for the cyclic peptide docking simulations with the ClusPro Dock protocol [22].

### 2.2. Cyclic Peptide-UEV Domain Protein Docking

The ClusPro Dock web-server were applied in the cyclic peptide-UEV domain receptors docking simulations [18,23]. Next, the possible cyclic peptide-UEV domain protein receptor complex structures were selected after sorting the weighted score values. The selected complex structures were then selected for subsequent equilibrium and production GaMD simulations.

### 2.3. The Detail Information of GaMD Simulations

**Figure 1:**
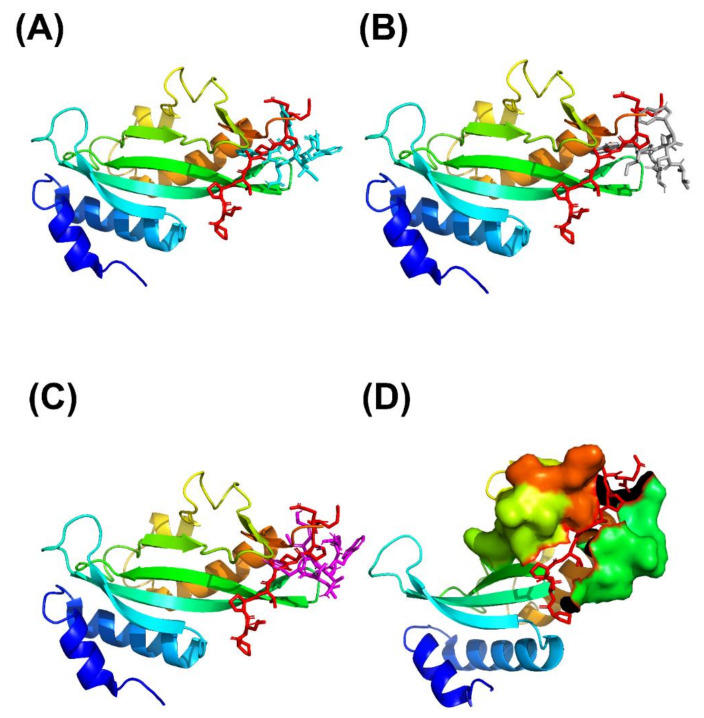
of the binding of UEV domain protein (ribbon model) with the native peptide shown as a stick model (red) and the three cyclic peptides shown as a stick model: (**A**) CP1 (cyan), (**B**) CP2 (gray), and (**C**) CP3 (magentas). (PDB ID: 3obu for the UEV domain protein with the native peptide) (**D**) The binding motif region of UEV domain protein (residue number: 61–66, 90–100 and 139–145) is shown as surface model, and the native peptide is shown as a stick model (red).

From the ClusPro peptide docking simulations, the complex structures with the lowest potential of mean force (PMF) values were selected and then inserted into TIP3P water molecules box (box size: approximately 7.14 × 9.21 × 7.80 nm^3^). These initial structures were chosen as refence structures for analyzing the C.M. distances and backbone RMSDs in our PMF profiles. These initial cyclic peptide-UEV domain protein structures were then simulated using the AMBER 18 software with the all-hydrogen amino acid AMBER FF99SB force field [24,25,26]. All initial cMD simulations were performed in the NPT ensembles with a constant temperature of 310 K, unless stated otherwise, using the Verlet integrator with an integration time step of 0.002 ps and SHAKE constraints [27] for all covalent bonds involving hydrogen atoms. For the long-range electrostatics, electrostatic interactions between molecules were performed using the particle mesh Ewald (PME) method [28], and the 2.0 nm cutoff distance was for van der Waals forces calculations. At first, these initial structures were minimized for 100,000 conjugate gradient steps and then performed with 1-ns NVT and 1-ns NPT cMD simulations. Additionally, the final structures from our cMD simulations were performed in 20-ns GaMD equilibration and 300-ns GaMD production simulations [29]. GaMD simulation trajectories were recorded every 0.2 ps for analyzing the C.M. distances and backbone RMSDs in our PMF profiles. Four times 300-ns GaMD production simulations were selected to analyz the C.M. distance and backbone RMSD of the cyclic peptides and UEV domain protein with the AmberTools cpptraj software [30]. For 2D PMF profiles, the PyReweighting toolkit was applied to reweight the four time GaMD each cyclic peptide–UEV domain protein systems [31]. The backbone RMSDs of UEV binding domain (residue numbers: 61–66, 90–100 and 139–145) and the distances (C.M) between the centers of the two domains (UEV domain protein residue numbers: 61–66, 90–100 and 139–145; the three cyclic peptides) (Figure 1D) were used as reaction coordinates. From our 2D PMF profiles, the cyclic peptide-VEU domain protein complex structures with the lowest PMF values (PMF is equal to zero) were applied with 10-ns cMD simulations for SIE free energy calculations and binding mode (hydrogen bonds and nonbonding interactions) analysis by using AmberTools cpptraj software [30]. Detailed information concerning GaMD and SIE are listed in the supporting information.

### 2.4. Gaussian Accelerated Molecular Dynamics Simulation (GaMD)

GaMD is a biasing method for enhanced sampling of biomolecular conformations by adding a harmonic boost potential to smooth the biomolecular potential energy surface [29]. Where V is a bio molecular system potential energy, E is a systemic referenced energy, and ΔV is a harmonic boost potential energy. If V > E, ΔV = 0. If V < E, a ΔV is applied in the biosystem as follows:

(1) $$\Delta V=\frac{1}{2}K\;{\left(E-V\right)}^{2},\;\mathrm{if}\;V<E$$

For GaMD simulations, the biased system potential energy (V*) is shown as

(2) $$V^{*}=V+\frac{1}{2}K\;{\left(E-V\right)}^{2},\mathrm{if}\;V<E$$

where K is a harmonic force constant

If V1 < V2, V1* < V2*. By replacing V* with Equation (2), the relationship is expressed as

(3) $$E<\frac{1}{2}\left(V1+V2\right)+\frac{1}{K}$$

If V1 > V2. By replacing V* with Equation (2), the relationship is expressed as

(4) $$E>\frac{1}{2}\left(V1+V2\right)$$

Combing Equations (3) and (4) is expressed as

(5) $$\mathrm{Vmax}\leq E\leq \mathrm{Vmin}+\frac{1}{K}$$

where Vmin and Vmax are the minimum and maximum potential energies.

From Equation (5), we can derive

(6) $$\frac{1}{K}\leq \frac{1}{\mathrm{Vmax}-\mathrm{Vmin}}$$

where K constant is defined as

(7) $$K=K0\left(\frac{1}{\mathrm{Vmax}-\mathrm{Vmin}}\right),\;0<K0\leq 1$$

K0 is the magnitude of the applied boost potential. Standard deviation (SD) of ΔV must be sufficiently small to ensure accurate reweighting [31].

(8) $$\sigma_{\Delta V}=\sqrt{{\left(\frac{\partial \Delta V}{\partial V}|V=\mathrm{Vave}\right)}^{2}\sigma_{V}{}^{2}}=K\;\left(E-\mathrm{Vave}\right)\sigma_{V}\leq \sigma_{0}$$

where the Vave and σV are the average and SD of the potential energies, respectively, and σΔV is the SD of ΔV with σ0 as a user-specified upper limit for accurate reweighting of potential energies. The SDs of the total potential and dihedral potential boosts are 10 kcal/mol each GaMD simulations.

If E = Vmax, we can derive Equation (5) and the result is expressed as

(9) $$K0\leq \frac{\sigma 0}{\sigma V}\frac{\mathrm{Vmax}-\mathrm{Vmin}}{\mathrm{Vmax}-\mathrm{Vave}}$$

According to Equations (6) and (7), K0 can be defined as

(10) $$K0=\min\left\{1.0,\frac{\sigma 0}{\sigma V}\frac{\mathrm{Vmax}-\mathrm{Vmin}}{\mathrm{Vmax}-\mathrm{Vave}}\right\}$$

If E = Vmin + 1/k, we can derive Equation (8) and the result is expressed as

(11) $$K0\geq \left(1-\frac{\sigma 0}{\sigma V}\right)\frac{\mathrm{Vmax}-\mathrm{Vmin}}{\mathrm{Vmax}-\mathrm{Vave}}$$

The GaMD boost potential (ΔV) is given as follows:

(12) $$\Delta V=\frac{1}{2}K0\frac{1}{\mathrm{Vmax}-\mathrm{Vmin}}{\left(E-V\right)}^{2},\;\mathrm{if}\;V<E$$

For our GaMD simulations, the magnitude K0 = 1.0, Vmax and Vmin were obtained through normal MD (cMD) simulations. 

### 2.5. SIE Free Energy Calculations

The binding free energies of the UEV domain protein with the three cyclic peptide inhibitors were calculated for 10 ns cMD simulations. The SIE [32] function is expressed as

(13) $$\Delta G_{bnd}\left(\rho ,D_{in},\;\alpha ,\gamma ,C\right)=\alpha \times \left[E_{c}\left(D_{in}\right)+\Delta G_{bind}^{R}\left(\rho ,D_{in}\right)+E_{vdw}+\gamma \Delta MSA\left(\rho \right)\right]+C$$

where *E_c_* and *E_vdw_* are the intermolecular electrostatic and van der Waals interaction energies, respectively. $\Delta G_{bind}^{R}$ is the reaction field energy between the bound states and unbond state, and $\Delta G_{bind}^{R}$ is solved by the Poisson equation [33,34]. ΔMSA is the change in the molecular surface area upon binding [35]. Additionally, the other parameters are as follows: α = 0.1048, Din = 2.25, ρ = 1.1, γ = 0.0129 kcal/(mol Å2), and C = −2.89 kcal/mol [36].

## 3. Results

### 3.1. Cyclic Peptide-VEU Domaim Protein Docking Simulations

**Figure 2:**
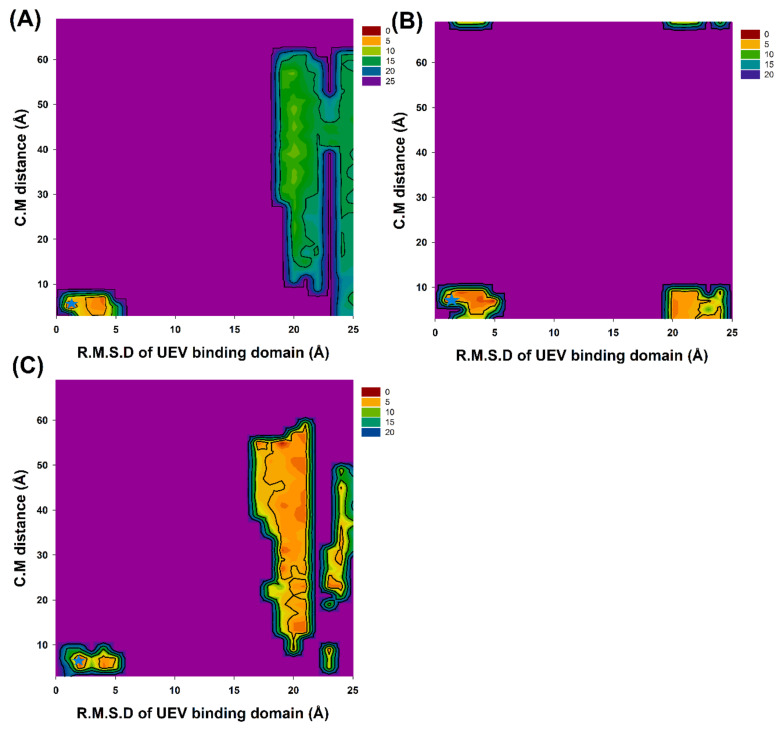
2D PMF profiles of the backbone RMSDs of UEV binding domain (residue numbers: 61–66, 90–100 and 139–145) and the distances between the centers of the two domains (UEV domain protein residue numbers: 61–66, 90–100 and 139–145; the three cyclic peptides): (**A**) CP1, (**B**) CP2 and (**C**) CP3. Stars mark (cyan) is at the lowest PMFs value of 2D PMF profiles.

These cyclic peptides were docked into the UEV domain protein structure. The initial cyclic peptide-UEV domain proteins complex structures were selected after sorting the weighted score values. Our cyclic peptide docking results were shown in Table 2 and Figure 2. From Table 2 and Figure 2, Our results showed that these cyclic peptides could bind to the motif of the UEV domain protein, and these initial complex structures were reasonable for further cMD and GaMD simulations.

### 3.2. Prediction of Cyclic Peptide Binding Conformations through 2D PMF Profiles

**Figure 3:**
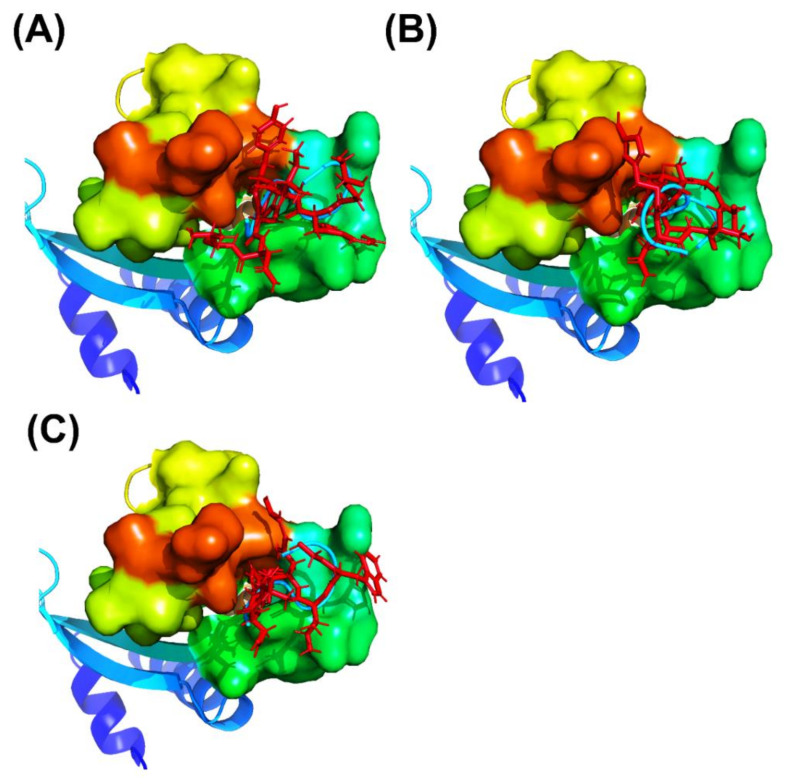
Overview of peptide docking conformations of the three cyclic peptides (cartoon model, cyan) obtained using ClusPro Dock are compared with the complex structures with the GaMD refinement (stick model, red): (**A**) CP1, (**B**) CP2, and (**C**) CP3.

GaMD simulations has been prove to refine the cyclic peptide-protein receptor complex structures. The C.M. distances and backbone RMSDs of the four times 300 ns-GaMD simulation trajectories were analyzed using AmberTools cpptraj software. The GaMD simulations were reweighted using the PyReweighting toolkitto calculate the 2D PMF profiles (Figure 2 and Table 3) Because the structure with the lowest PMF values might be reasonable and possible, the chosen complex structures were selected for SIE free energy calculations, and confirmed with experimental free energies. For the CP1 simulation system, the lowest PMFs were at the UEV binding domain backbone RMSD of 1.0 and the C.M. distance of 5.0 Å in the GaMD simulations, respectively. For the CP2 simulation system, lowest PMFs were at the UEV binding domain backbone RMSD of 2.0 and the C.M. distance of 9.0 Å. For the CP3 simulation system, the lowest PMFs were at the UEV binding domain backbone RMSD of 2.0 and the C.M. distance of 7.0 Å. When the cyclic peptide docking simulations were compared with GaMD refined structures, significant conformational changes and the reduced PMF values were observed in UEV domain protein during cyclic peptide binding (Figure 3 and Table 3). These GaMD refined structures were selected to use in 10-ns cMD simulations for SIE free energy calculations and binding mode analysis. From the 2D PMF profiles and Table 3, GaMD simulations significantly refined the three cyclic peptide-UEV domain docking conformations.

### 3.3. SIE Free Energy Calculations

The predicting and experimental binding free energies (ΔG) of the three cyclic peptides with UEV domain protein are presented in Table 4 and Table 5. For the three cyclic peptides, the predicted ΔG values are −6.05, −5.31 and −6.52 kcal/mole, respectively. For the predicting binding free energies erroneous analysis, the error of our predicting binding free energies (ΔGdiff) are −0.51, 0.314 and 0.020 kcal/mol, respectively. Therefore, the GaMD refined complex structures could present a satisfactory correlation between the predicting and experimental binding free energies of all three cyclic peptides. Moreover, GaMD refined complex structures could provide more accurate and reasonable predictions.

### 3.4. Analysis of the Binding Modes of UEV Domain Protein with the Three Cyclic Peptide Inhibitors

**Figure 4:**
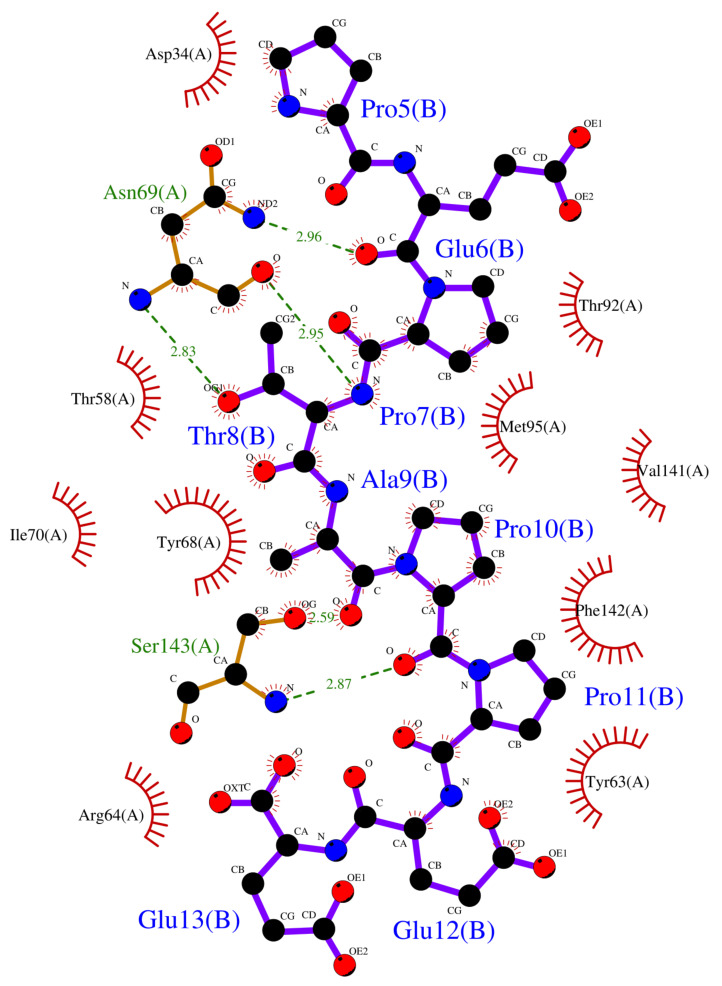
Overview of binding modes of the UEV domain proteins with the native linear peptide.

**Figure 5:**
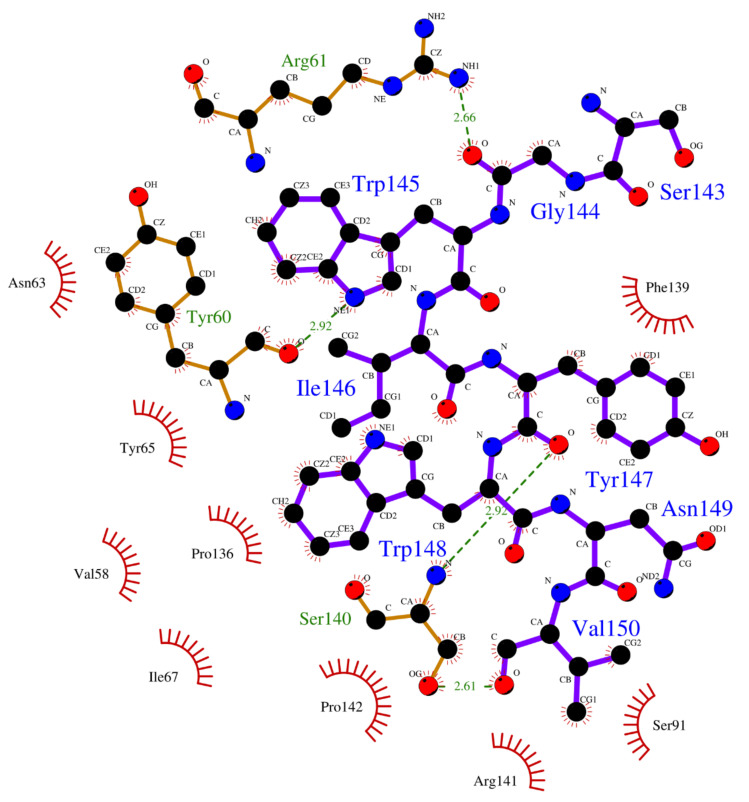
Overview of binding modes of the UEV domain proteins with CP1.

**Figure 6:**
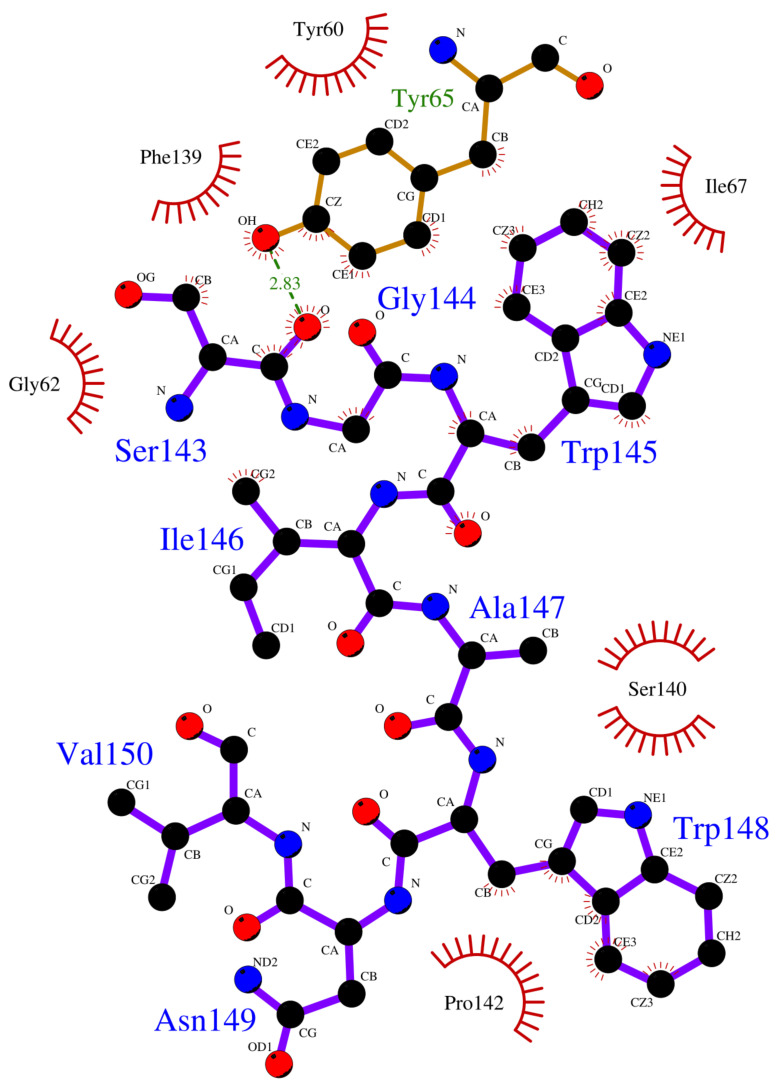
Overview of binding modes of the UEV domain proteins with CP2.

**Figure 7:**
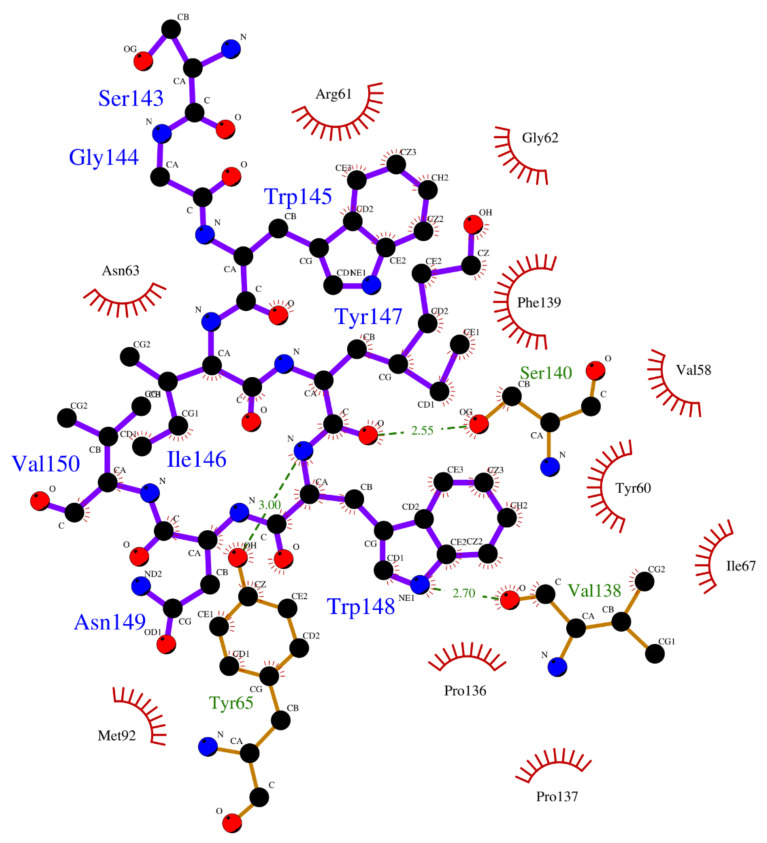
Overview of binding modes of the UEV domain proteins with CP3.

Crystallographic structures revealed the binding modes of the UEV domain proteins with the native linear peptide, and this binding mode is shown in Figure 4. In the UEV domain protein-native peptide complex, the native peptide residues Glu6, Pro7, Thr8 and Pro10 can form hydrogen bonds with the Asn69 and Ser143 residues of UEV domain protein, whereas the native peptide residues Pro5, Glu6, Pro7, Thr8, Ala9, Pro10, Pro11 and Glu13 have nonbonding interactions with the Asp34, Thr58, Tyr63, Arg64, Tyr68, Ile70, Thr92, Met95, Val141 and Phe142 residues of UEV domain protein. For analyzing the binding modes of the three cyclic peptides-UEV domain proteins, the 10-ns cMD simulations (SIE free energies calculation trajectories) were applied in the analysis. For finding out the hot-spot residues, the binding mode analysis of our 10-ns cMD simulations, which are more than a fraction of 30%, are shown in Table 6. The binding mode analysis of the UEV domain protein with the three cyclic peptides through ClusPro Dock simulations are shown in Figure 5, Figure 6, Figure 7. In the UEV domain protein-CP1 simulations, the CP1 residues Gly144, Trp145, and Tyr147 can form hydrogen bonds with the Arg61, Tyr60, and Ser140 residues of UEV domain protein, whereas the CP1 residues Trp145, Tyr147, and Trp148 have nonbonding interactions with the Asn63, Pro142, Pro139, Pro136, and Ile67 residues of UEV domain protein. In the UEV domain protein-CP2 simulations, the CP2 residue Gly144 can form hydrogen bonds with the Tyr65 residues of UEV domain protein, whereas the CP2 residues Trp145, Ser143, and Trp148 have nonbonding interactions with the Tyr60, Phe139, Pro142, and Gly62 residues of UEV domain protein. In the UEV domain protein-CP3 simulations, the CP3 residues Tyr147 and Trp148 can form hydrogen bonds with the residues Tyr65, Ser140, and Val139 residues of UEV domain protein, whereas the CP3 residues Trp145, Tyr147, Ile146, and Trp148 have nonbonding interactions with the Arg61, Gly62, Phe139, Val58, Asn63, Ile67, Pro136, and Pro137 residues of UEV domain protein. Moreover, these binding modes analysis could reveal the essential residues of the three cyclic peptides in the cyclic peptides-UEV domain proteins interactions.

## 4. Discussion

Our validation of the Tsg101 UEV protein and the linear peptide (amino sequence: PEPTAPPEE and PDB ID: 3obu) is reasonable and shown in the supporting information. We are confident about our predictions. Cyclic peptides are a yet underexploited candidates for drug discovery. Compared to their linear counterparts, cyclic peptides have enzymatic resistance and lower 3D conformational freedom, which allow them to bind to protein receptors with higher binding affinity and specificity [37]. For speeding up the cyclic peptides drug discovery, many peptide-protein computational docking approaches are developed to address this issue. Unfortunately, most peptide docking approaches remain a great *challenge* for most computational docking programs because of the peptide flexibility. Even though these docking methods are fast, these methods still lack thermodynamic refinements and cannot provide accurate peptide binding conformational predictions. These peptide docking methods can’t perform docking simulations with cyclic peptides and can only perform related simulations with linear peptides [16,38,39]. To overcome this issue, we used an approach combined global peptide docking and GaMD methods to investigate the cyclic peptides-protein receptors interactions. This approach has been confirmed to succeed in predicting the linear peptide–protein binding conformations [13]. Compared to their linear counterparts, cyclic peptides indeed reduce 3D conformational freedom [40]. Therefore, this approach is suitable for studying the interactions between the three cyclic peptides and UEV domain proteins.

GaMD refinement simulations revealed the 2D PMF profiles (Figure 2 and Table 3), and we determined the most possible and reasonable complex structures with the lowest PMF values. Next, the 10-ns normal MD simulations were applied for SIE free energy calculations and the cyclic peptides-VEU domain protein binding mode analysis. Our predicting binding free energies have been confirmed to succeed in the three cyclic peptides binding conformational predictions. Therefore, we believe that our predictions could provide a reasonable and accurate insight into the interactions of UEV domain proteins with the three cyclic peptides. Since the mot of peptides can bind to the largest pocket on protein receptor surfaces, the complex aspect of the peptide binding still remains challenging. When our predictions were compared with the UEV domain protein experimental information (Table 4, Table 5, Table 6 and Figure 4, Figure 5, Figure 6, Figure 7), the three cyclic peptide binding modes were found to be very different from the native peptide-UEV domain protein binding information. The cyclic CP1 and CP3 peptides can prevent the binding of UEV domain protein to P6 motif, thereby stopping HIV and Ebola virus budding [41]. The cyclic CP2 peptides indeed have weaker binding ability with UEV domain proteins.

Since the single point residue mutations of peptides might have different 3D conformations and affect the binding affinities with protein receptors [42,43]. The two cyclic peptides (CP2 and CP3) are the single residue mutation of the CP1 peptide. Therefore, we analyzed the binding modes of the cyclic peptides with UEV domain protein and found out the hot-spot residues each the cyclic peptide. Regarding the CP1-UEV domain protein interactions, the residues Gly144, Trp145, and Tyr147 of CP1 can form hydrogen bonds with UEV domain proteins, and residues Trp145, Tyr147, and Trp148 of CP1 can interact with UEV domain proteins via the nonbonding interactions. Regarding the CP2–UEV domain protein interactions, the Gly144 residue of CP2 can form hydrogen bonds with UEV domain proteins, and Trp145, Ser143, and Trp148 residues of CP2 can interact with UEV domain proteins via the nonbonding interactions. Regarding the CP3–UEV domain proteins interactions, our results show that the Tyr147 and Trp148 residue of CP3 can form hydrogen bonds with UEV domain proteins, and Trp145, Tyr147, Ile146, and Trp148 residues of CP3 can interact with UEV domain proteins via the nonbonding interactions. Compared with the three cyclic peptides, the mutation of Tyr147 can reduce UEV domain protein binding abilities. To sum up, the residues Trp145, Tyr147, and Trp148 of the native cyclic peptide (CP1) indeed play essential roles in the cyclic peptides–UEV domain proteins interactions.

## 5. Conclusions

For speeding up the cyclic peptides drug discovery, an approach, which combins global docking, normal MD simulations, GaMD simulations, 2D free energy profiles (2D PMFs), and SIE free energy techniques, was applied in studying the interactions between the three cyclic peptides and UEV domain proteins. From the GaMD refinement simulations, our 2D PMF profiles found out the most possible cyclic peptide-UEV domain protein complex structures. Then our SIE binding free energy predictions were close to the experimental binding free energies, and these results confirmed that our cyclic peptide binding conformational predictions seemed reasonable and possible. Next, we analyzed the binding modes of the cyclic peptides with UEV domain protein from 10-ns cMD simulations each cyclic peptide. The hot spot residues Trp145, Tyr147, and Trp148 of the three cyclic peptides can influence UEV domain protein binding affinities. Our study not only can increase the accuracy of cyclic peptide–protein 3D conformational prediction, but this study also can help future cyclic peptide drug discovery. Advancements in computational methods and computing power are expected to further aid in addressing the challenges in cyclic peptide drug design.

## Figures and Tables

**Table 1:** Inhibitors of UEV domains.

Peptide Inhibitors [10]		Experimental Binding Free Energies Values (ΔGexp)(kcal/mol)
Native	Pro-Glu-Pro-Thr-Ala-Pro-Pro-Glu-Glu	−6.05 (IC_50_ = 54.0 uM),
CP1	Cyclo-(Ser-Gly-Trp-Ile-Tyr-Trp-Asn-Val)	−6.11 (IC_50_ = 48.6 uM),
CP2	Cyclo-(Ser-Gly-Trp-Ile-Ala-Trp-Asn-Val)	−5.00 (IC_50_ = 296.3 uM),
CP3	Cyclo-(Ser-Gly-Trp-Ile-Tyr-Trp-Ala-Val)	−6.50 (IC_50_ = 25.8 uM),

ΔGexp: experimental free energies (kcal/mol).

**Table 2:** Of cyclic peptide binding interactions for ClusPro docking simulations.

Cyclic Peptide Inhibitors	Weighted Score (kcal/mol)
CP1: Cyclo-(Ser-Gly-Trp-Ile-Tyr-Trp-Asn-Val)	−705.60
CP2: Cyclo-(Ser-Gly-Trp-Ile-Ala-Trp-Asn-Val)	−641.20
CP3: Cyclo-(Ser-Gly-Trp-Ile-Tyr-Trp-Ala-Val)	−752.40

**Table 3:** D PMF profile information of the cyclic peptide–UEV domain complex structure.

Cyclic Peptides	Peptide Docking Simulation			GaMD Refinement		
	RMSD (Å)	C.M. (Å)	PMF (kcal/mol)	RMSD (Å)	C.M. (Å)	PMF (kcal/mol)
CP1	2.5	8.6	14.6	1.0	5.0	0.0
CP2	1.5	10.4	11.5	2.0	9.0	0.0
CP3	1.7	9.8	14.3	2.0	7.0	0.0

**Table 4:** Free energy prediction results of cyclic peptides for inhibiting UEV domain protein.

Cyclic Peptides	UEV Domain Protein		
	ΔGexp (Kcal/mol)	ΔGcal (Kcal/mol)	ΔGdiff (Kcal/mol)
CP1	−6.11	−6.05	−0.051
CP2	−5.00	−5.31	0.314
CP3	−6.50	−6.52	0.020

ΔG_exp_: Experimental Free Energies; ΔG_cal_: Predicting Free Energies; ΔG_diff_ = ΔG_exp_ − ΔG_cal_ (kcal/mol).

**Table 5:** Information of SIE free energy calculations for UEV domain protein.

Cyclic Peptides	CP1	CP2	CP3
Inter vdW	−32.54 ± 0.41	−28.16 ± 0.17	−31.42 ± 0.42
Inter Coulomb	−41.37 ± 0.15	−38.45 ± 0.23	−45.94 ± 0.13
Reaction Field	64.32 ± 0.81	62.11 ± 0.15	62.54 ± 0.26
Cavity	−20.68 ± 0.17	−18.64 ± 0.13	−19.85 ± 0.19
Constant	−2.89	−2.89	−2.89
△G_Binding_ (kcal/mol)	−6.05 ± 0.14	−5.31 ± 0.16	−6.52 ± 0.11

**Table 6:** Binding modes of UEV domain protein with the cyclic peptides.

Case	Hydrogen Bonds					Non-Bonding Interactions
	Acceptor	Donor	Fraction (%)	Average Distance (Å)	Average Angle (θ)	Residues Fraction (%)
CP1	Gly144	Arg61	67.30	2.65	144.21	Trp145- Asn63 (54%) Tyr147-Pro142 (35%) Tyr147-Phe139 (41%) Trp148-Pro136 (32%) Trp148-Ile67 (36%)
	Tyr60	Trp145	66.10	2.91	147.33	
	Ser140	Tyr147	64.40	2.89	151.14	
CP2	Gly144	Tyr65	42.51	2.85	158.13	Trp145- Tyr60 (41%) Ser143-Phe139 (32%) Trp148-Pro142 (48%) Ser143- Gly62 (45%)
CP3	Tyr65	Tyr147	68.31	2.91	151.64	Trp145-Arg61 (42%) Trp145-Gly62 (31%) Tyr147-Phe139 (45%) Tyr147-Val58 (34%) Ile146-Asn63 (30%) Trp148-Ile67 (41%) Trp148-Pro136 (36%) Trp148-Pro137 (39%)
	Tyr147	Ser140	61.25	2.49	144.32	
	Val138	Trp148	74.34	2.65	149.33	

Number of amino acid: 1–142 is UEV domain protein and 143–150 is the cyclic peptides.

## Supplementary Materials

The following are available online at https://www.mdpi.com/2073-4360/12/10/2235/s1. We followed our strategy to simulate the refinement of the Tsg101 UEV protein and the linear peptide (amino sequence: PEPTAPPEE). Then our refinement was compared with the x-ray structure (PDB ID: 3obu). Our results are shown in Figures S1−3. Figure S1: The linear peptide (amino sequence: PEPTAPPEE) is build by VEGAZZ software, Figure S2: 2D PMF profiles of the backbone RMSDs of UEV binding domain (residue numbers: 61-66, 90-100 and 139-145) and the distances between the centers of the two domains (UEV domain protein residue numbers: 61-66, 90-100 and 139-145; the linear peptide), Figure S3: Overview of the binding of UEV domain protein (ribbon model) with the native peptide shown as a stick model (red), the cluspro docking result shown as stick model (cyan), and the our final result shown as stick model (green).
